# Investigating the effects of frailty on six-month outcomes in older trauma patients admitted to UK major trauma centres: a multi-centre follow up study

**DOI:** 10.1186/s13049-023-01169-8

**Published:** 2024-01-04

**Authors:** Elaine Cole, Robert Crouch, Mark Baxter, Chao Wang, Dhanupriya Sivapathasuntharam, George Peck, Cara Jennings, Heather Jarman

**Affiliations:** 1 Centre for Trauma Sciences, Queen Mary University, London, England; 2 University Hospital Southampton NHS Foundation Trust, Southampton, England; 3 Kingston University, Kingston, England; 4 Barts Health NHS Trust, London, England; 5 Imperial College Healthcare NHS Trust, London, England; 6 King’s College Hospital NHS Foundation, Kingston, England; 7 St George’s University Hospital NHS Foundation Trust, London, England

**Keywords:** Frailty, Older Trauma, Geriatric trauma, Health-related quality of life, Outcomes.

## Abstract

**Background:**

Pre-injury frailty is associated with adverse in-hospital outcomes in older trauma patients, but the association with longer term survival and recovery is unclear. We aimed to investigate post discharge survival and health-related quality of life (HRQoL) in older frail patients at six months after Major Trauma Centre (MTC) admission.

**Methods:**

This was a multi-centre study of patients aged ≥ 65 years admitted to five MTCs. Data were collected via questionnaire at hospital discharge and six months later. The primary outcome was patient-reported HRQoL at follow up using Euroqol EQ5D-5 L visual analogue scale (VAS). Secondary outcomes included health status according to EQ5D dimensions and care requirements at follow up. Multivariable linear regression analysis was conducted to evaluate the association between predictor variables and EQ-5D-5 L VAS at follow up.

**Results:**

Fifty-four patients died in the follow up period, of which two-third (64%) had been categorised as frail pre-injury, compared to 21 (16%) of the 133 survivors. There was no difference in self-reported HRQoL between frail and not-frail patients at discharge (Mean EQ-VAS: Frail 55.8 vs. Not-frail 64.1, *p* = 0.137) however at follow-up HRQoL had improved for the not-frail group but deteriorated for frail patients (Mean EQ-VAS: Frail: 50.0 vs. Not-frail: 65.8, *p* = 0.009). There was a two-fold increase in poor quality of life at six months (VAS ≤ 50) for frail patients (Frail: 65% vs. Not-frail: 30% *p* < 0.009). Frailty (β-13.741 [95% CI -25.377, 2.105], p = 0.02), increased age (β -1.064 [95% CI [-1.705, -0.423] *p* = 0.00) and non-home discharge (β -12.017 [95% CI [118.403, 207.203], *p* = 0.04) were associated with worse HRQoL at follow up. Requirements for professional carers increased five-fold in frail patients at follow-up (Frail: 25% vs. Not-frail: 4%, *p* = 0.01).

**Conclusions:**

Frailty is associated with increased mortality post trauma discharge and frail older trauma survivors had worse HRQoL and increased care needs at six months post-discharge. Pre-injury frailty is a predictor of poor longer-term HRQoL after trauma and recognition should enable early specialist pathways and discharge planning.

## Background

Traumatic injury is a substantial health burden for older patients with a significant proportion having ongoing functional and psychological difficulties long after hospital discharge [1]. Populations are ageing, and there is a corresponding increase in the proportion of older trauma patients [2]. Trauma systems are also better at identifying previously under-recognised populations of older trauma patients [3]. Accordingly, trauma systems have adapted their practice and guidance for older patients, focussing on differing mechanisms of injury and age-related physiological variations, to ensure timely assessment and management in the early phases of care [4, 5]. However older trauma patients are a heterogeneous group in relation to age, pre-injury functional status and comorbidities, all of which might impact on longer-term recovery [6]. Recent evidence suggests that in older trauma patients, frailty rather than chronological age is strongly associated with in-hospital and 30-day mortality and adverse discharge disposition [2, 7, 8]. Frailty is a state of reduced physiological and cognitive reserve and may affect an individual’s ability to respond to a stressor event such as traumatic injury [9]. Between six and twelve months post trauma-discharge, frail patients are also more likely to have a trauma-related readmission [10] and poor functional status [11], yet the effect of frailty on longer term survival and quality of recovery is not widely reported.

Characterising pre-injury frailty may help to support prognostication in the acute phases of trauma care [12, 13] but it may also identify those who are vulnerable to longer term mortality, poor recovery and reduced quality of life. Health related quality of life (HRQoL) describes an individual’s perception of their physical and mental health, cognitive and emotional status [14]. Patient reported HRQoL measures are used to quantify the effect of injury on health, assessing functional, psychological, social and mobility dimensions to provide an insight into recovery [15]. There are a variety of HRQoL measures and in the UK the Euroquol EQ-5D-5 L measure has been implemented within a national programme of patient-reported outcomes after major trauma for all ages of adult patients [16]. A recent systematic review identified that EQ-5D-5 L had good feasibility and application when administered to older people, including when support to complete was required [17]. Understanding HRQoL following trauma is also important in planning and supporting ongoing care, and to ensure that patients and families or carers are given realistic information on their expected recovery [14]. In older, frail trauma patients HRQoL appears to deteriorate after discharge from hospital. At 30 days after injury, frailty is reported to negatively impact on the recovery of health-related quality of life compared to the discharge baseline [18]. Beyond this time-point, the effects of frailty on longer-term HRQoL after trauma are not widely reported. Frail survivors of a trauma admission may suffer a markedly worse longer-term outcome and HRQoL compared with those who are not frail. Therefore, we aimed to investigate health related quality of life at six months following discharge from hospital, in older trauma survivors with and without pre-injury frailty. We also aimed to compare longer-term care needs between these two patient groups.

## Methods

This research comprised the longer-term outcome evaluation of the ‘Frailty in Major Trauma’ (FRAIL-T) multi-centre prospective observational study, carried out at five Major Trauma Centres (MTCs, Level 1 equivalent hospitals) in England [19]. The study was approved by the UK Social Care Research Ethics Committee (REC: 19/IEC08/0006).

Eligibility criteria included participants aged 65 or over, requiring trauma team activation and subsequently admitted to an MTC, irrespective of their injury severity score. All MTCs use a tiered trauma team activation (TTA) system depending on whether a full trauma team is required or not. Patients were approached for enrolment regardless of the level of TTA. Pre-injury frail status was determined during the in-patient phase of FRAIL-T and confirmed by geriatricians using the Clinical Frailty Scale (Frail defined as Clinical Frail Scale ≥ 6) [19]. Participants were consented for follow-up at the time of enrolment into the study. If a participant lacked capacity, then in line with the Health Research Authority for England and Wales guidance, a personal consultee who was able to advise on whether the patient should be included was approached. Quality of life data were collected via a questionnaire at two time points: on the day of discharge from hospital and at follow up, namely six months following discharge from hospital. When providing consent participants were asked to state their preference for receiving a follow-up questionnaire either by post or email, or administered over the telephone by a researcher trained in undertaking telephone interviews. Prior to follow-up at six-months the patient’s survival status was checked via the national digital record system (National Health Service Spine) to ensure it was appropriate to make contact. Participants who were known to be alive at follow up but who did not respond to the initial request were contacted by telephone up to three times, at which point if there no response they were assumed ‘lost to follow-up’.

The primary outcome was patient reported health related quality of life (HRQoL) at follow up compared to hospital discharge. HRQoL was assessed using two elements: the Euroqol EQ-VAS (visual analogue scale) which rates overall health on 0–100 scale where endpoints are labelled ‘The worst health you can imagine’ through to ‘The best health you can imagine’ [20]. A poor HRQoL was defined as a VAS rating ≤ 50 [21, 22]. Descriptions of the individual’s health status was measured using the EQ-5D-5 L system, a preference-based measure of self-reported health, which has been successfully validated in geriatric populations [23] and in frail older patients [24]. EQ-5D-5 L consists of five dimensions: mobility, self-care, usual activities, pain and discomfort, and anxiety and depression, and respondents report each dimension as *no problems, slight problems, moderate problems, severe problems* and *extreme problems* (or unable to complete) [25]. It has been developed for use either in face-to-face or telephone/online interviews when respondents are capable of self-reporting their health-related quality of life but unable to self-complete a paper/digital questionnaire [21]. We also recorded patient reported alteration in dependence status and care needs at follow up.

On day of hospital discharge participants were approached by a research team member and asked to complete the questionnaire. If a patient lacked capacity at follow-up, a relative or proxy (if available) was asked to complete the questionnaire on their behalf [26]. At six-month follow-up participants or their proxy were either sent an HRQoL for self-completion or contacted by telephone to complete the interviewer administered version of the tool.

Data were analysed using Stata (version 16.1). Continuous data were tested for normality using Shapiro Wilk tests. Parametric comparisons were conducted using t-tests or ANOVA with Tukeys post hoc testing, and reported as mean (standard deviation). Non-parametric comparisons were tested using Mann Whitney U or Kruskall Wallis tests with Dunn post hoc testing, and reported as median (interquartile range). All tests were two-sided. Analysis of categorical data was conducted using Fisher’s exact test and reported as number and percentage. The five EQ-5D-5 L dimensions were dichotomised into ‘no problems’ and ‘any problems’ [20] for comparison between cohorts at discharge and follow up. Multivariable linear regression analysis was conducted to evaluate the association between EQ-5D-5L VAS at follow up and predictor variables including frailty, age, sex, comorbidities, mechanism of injury, critical care admission, injury severity, length of stay and discharge destination. Robust standard errors were used and a p-value of < 0.05 was considered statistically significant. Potential non-linear effects of predictors were explored using fractional polynomial terms. Model specification was also checked by the link test and Ramsey’s regression specification-error test. Missing data was addressed using maximum likelihood method [27].

## Results

Of the 337 FRAIL-T study patients who survived hospital admission, 150 patients (45%) were not included in the follow up due to: no response ‘loss to follow up’ (119), not on NHS central record system to check survival status (10), did not consent to follow up (10) or unable to complete the HRQoL due to communication difficulties (10). Fifty-four patients (16%) died post discharge, leaving 133 included in the longer-term analysis (Table 1).

**Table 1:** Patient characteristics and outcomes

	Died post discharge (n = 54)	Not included in follow up (n = 150)	Included in follow up (n = 133)	*p*-value
**Pre-injury frailty**	32 (64%)	55 (37%)	21 (16%)	< 0.01
**Age^**	83.0 (7.8)	80.3 (8.3)	77.7 (7.5)	< 0.01**
**Female**	32 (59%)	80 (53%)	69 (52%)	0.85
**Predominant mechanisms of injury**:				0.01
***Fall < 2 m***	38 (70%)	77 (51%)	72 (54%)	
***Fall ≥ 2 m***	12 (22%)	42 (28%)	19 (14%)	
***Vehicular incident***	2 (4%)	24 (16%)	34 (26%)	
**Admission GCS^**	15 (14–15)	15 (14–15)	15 (15–15)	0.07
**Injury Severity Score~**	13 (9–20)	13 (9–20)	13 (9–17)	0.63
**Critical care admission**	6 (13%)	29 (22%)	18 (16%)	0.27
**Hospital LOS (days)~**	17 (7–27)	14 (5–20)	10 (4–18)	0.02*
**Pre-injury residential status**:				0.29
***Own accommodation***	47 (87%)	136 (91%)	126 (96%)	
***Residential care***	5 (5%)	13 (9%)	5 (4%)	
***Warden controlled accommodation***	2 (4%)	1 (1%)	1 (1%)	
**Discharge to home**	30 (56%)	105 (70%)	102 (77%)	< 0.01
**Discharge Eq. 5D VAS^**	53.9 (24.1)	59.1 (23.0)	61.8 (20.0)	0.16

^mean (standard deviation) or ~ median (Interquartile range) unless otherwise stated. Frail = Clinical Frailty Scale > 5 in hospital by ED or Geriatrician teams. GCS: Glasgow Coma Scale; LOS: Length of Stay, Eq. 5D VAS: Visual Analogue Scale. p value indicates comparison across groups (ANOVA / Kruskall Wallis or Chi Squared tests). ** signifies *p* < 0.01 when comparing age of patients who died and those who were alive and included (Tukeys post-hoc test). ). *signifies *p *= 0.04 when comparing LOS of patients who died with those who were alive and included (Dunn post-hoc test)

There was a four-fold increase in pre-injury frailty in those who died after discharge compared to those alive and included in the follow-up (Frail died: 64% vs. Frail not followed-up: 37% vs. Frail followed-up: 16%, *p* < 0.01). Patients who died prior to follow up were older (Died: 83 years vs. Not followed-up: 80 years vs. Followed-up: 77 years, *p* < 0.01), had experienced longer hospital stays (Died: 17 days vs. Not followed-up: 12 days vs. Followed-up: 10 days, *p* = 0.02) and were less likely to go directly to home from the MTC (Died: 56% vs. Not followed-up: 70% vs. Followed-up: 77%, *p* < 0.01).

Less than a fifth of the followed-up patients (16%) had been categorised as frail whilst in hospital according to the clinical frailty scale [28] (Table 2). Only half of frail patients were able to complete the follow up independently compared to 92% of the not-frail group. Frail patients alive at follow up were older (Frail: 79 years vs. Not-frail 77 years, *p* = 0.04), had a greater number of co-morbidities (Frail: 3.3 vs. Not-frail: 2.1, *p* < 0.01) and the majority sustained injury during a low-level fall (Frail: 76% vs. Not-frail: 50%, *p* = 0.03). There were no differences in re-admission rates between frail and not-frail patients during the post-discharge period (Table 2).

**Table 2:** Frail vs. Not-frail patients characteristics and outcomes

	Frail (n = 21)	Not-frail (n = 112)	*p*-value
**Follow up completed by patient**	10 (50%)	103 (92%)	-
**Age^**	79.2 (6.9)	77.0 (7.2)	0.04
**Female**	14 (67%)	55 (49%)	0.15
**n. co-morbidities^**	3.3 (1.5)	2.1 (1.3)	< 0.01
**Fall < 2 m**	16 (76%)	56 (50%)	0.03
**Fall ≥ 2 m**	3 (14%)	16 (14%)	0.99
***Vehicular incident***	1 (5%)	33 (29%)	0.01
**Injury Severity Score~**	13 (9–20)	13 (9–17)	0.53
**Critical care admission**	1 (5%)	17 (15%)	0.30
**Hospital LOS (days)~**	14 (2–20)	9 (4–17)	0.95
**Pre-injury residential status**:			0.11
***Own accommodation***	18 (86%)	109 (97%)	
***Residential care***	2 (10%)	3 (3%)	
***Warden controlled accommodation***	1 (5%)	0 (0%)	
**Discharge to home**	15 (71%)	87 (78%)	0.57
**Hospital re-admission**	7 (33%)	22 (20%)	0.13
**n. re-admissions^**	1.17 (0.41)	1.50 (0.83)	0.35

^mean (SD) or ~ median (interquartile range) unless otherwise stated. Frail = Clinical Frailty Scale > 5 in hospital by ED or Geriatrician teams. LOS: Length of Stay. p value indicates comparison between frail and not-frail patients

**Fig. 1:**
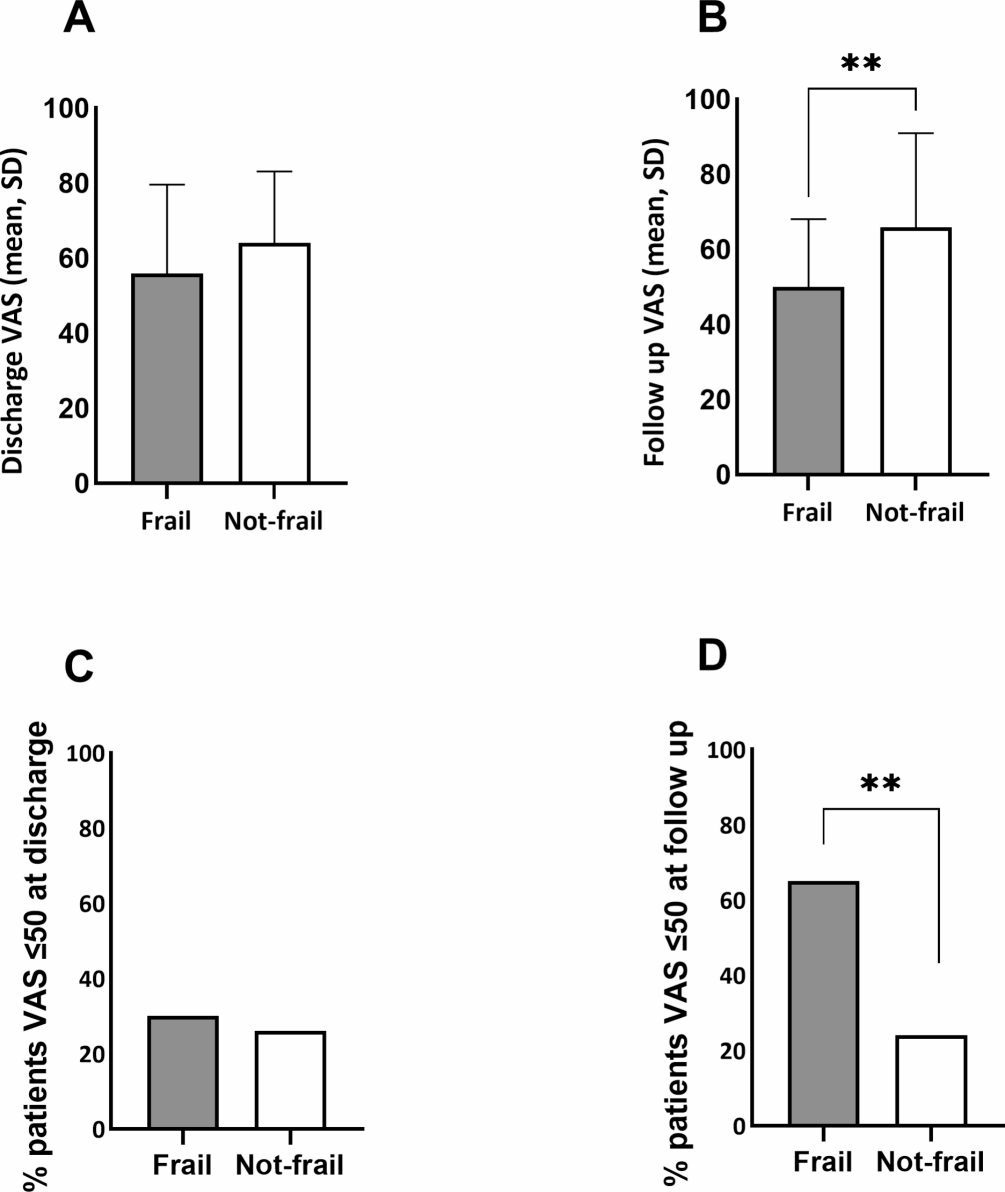
Self-rated health related quality of life (HRQoL). **(A)** Visual analogue scores (VAS) (Mean, Standard Deviation) **at hospital discharge**: **Frail** 55.8 (42.7–69.0) vs. **Not-frail** 64.1 (60.0–68.1), *p* = 0.137. **(B)** VAS (Mean, Standard Deviation) **at follow up**: **Frail** 50.0 (41.3–58.6) vs. **Not-frail** 65.8 (61.0–70.7), *p* < 0.01 (T-tests) **(C)** Percentage patients with a VAS ≤ 50 at discharge: **Frail** 30% vs. **Not-frail** 26%, *p* = 0.784. (D) Percentage patients with a VAS ≤ 50 at follow up: **Frail** 65% vs. **Not-frail** 25%, *p* < 0.009 (Fishers exact test)

At discharge, self-reported health related quality of life (HRQoL) was similar between groups (VAS: Frail: 55.8 vs. Not-frail: 64.1, *p* = 0.137, Fig. 1A). Whereas at follow-up, those who were not frail had improved, compared to a deterioration from discharge baseline in frail patients (VAS: Frail: 50 vs. Not-frail: 65.8, *p* < 0.01, Fig. 1B). The percentage of those reporting poor quality of life (VAS ≤ 50) at discharge was comparable between groups (Fig. 1C), but at follow up there was a two-fold increase in poor HRQoL for frail patients (Frail: 65% vs. Not-frail: 30% *p* < 0.01, Fig. 1D). In multivariable regression the presence of frailty (β-13.741 [95% CI -25.377, -2.105], *p* = 0.02), increased age (β -1.064 [95% CI [-1.705, -0.423] *p* = < 0.01) and non-home discharge (β -12.017 [95% CI [118.403, 207.203], *p* = 0.04) were associated with a poor quality of life at follow up (Table 3).

**Table 3:** Factors associated with Health Related QoL (Eq. 5D VAS) at follow up

	Beta coefficient	95% Confidence Intervals	*p*-value
**Frail CFS > 5 based on in-hospital score**	-13.741	[-25.377, -2.105]	0.02
**Age**	-1.064	[-1.705, -0.423]	< 0.01
**Sex (Female = ref)**	-5.083	[-14.518, 4.351]	0.29
**Number of comorbidities**	-0.608	[-4.487, 3.272]	0.76
**Mechanism of injury (Others = ref)**			
**Fall < 2 m**	3.237	[-12.402, 18.876]	0.68
**Fall from ≥ 2 m**	3.010	[-13.845, 19.865]	0.72
**Critical care admission**	1.304	[-13.260, 15.867]	0.86
**Length of stay in Major Trauma Centre**	0.182	[-0.242, 0.606]	0.40
**Transfer to other care (Discharge to home = ref)**	-12.017	[-23.232, -0.802]	0.04
**Intercept**	162.803	[118.403, 207.203]	< 0.01

R-squared = 0.194. Injury severity was removed from the final model due to missing data and a further analysis using maximum likelihood method: *p* = 0.392

**Fig. 2:**
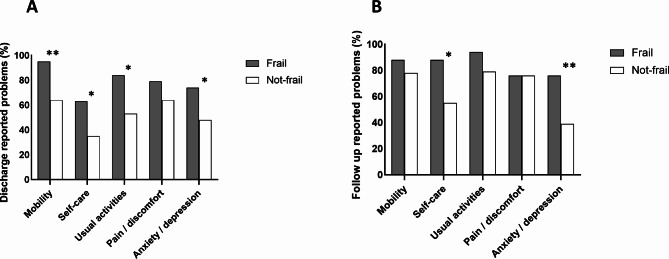
Percentage of reported problems in EQ-5D-5 L dimensions for frail and not-frail patients at **(A) Discharge.** Mobility: Frail 95% vs. Not-frail 64%, *p* < 0.01, Self-care: Frail 63% vs. Not-frail 35%, *p* = 0.03, Usual activities: Frail 84% vs. Not-frail 53%, *p* = 0.01, Pain: Frail 79% vs. Not-frail 64%, *p* = 0.299, Anxiety/depression: Frail 74% vs. Not-frail 48%, *p* = 0.04. **(B) Follow-up.** Mobility: Frail 88% vs. Not-frail 78%, *p* = 0.511, Self-care: Frail 88% vs. Not-frail 55%, *p* = 0.01, Usual activities: Frail 94% vs. Not-frail 79%, *p* = 0.189, Pain: Frail 76% vs. Not-frail 76%, *p* = 0.999, Anxiety/depression: Frail 76% vs. Not-frail 39%, *p* < 0.01. (Chi squared tests)

**Fig. 3:**
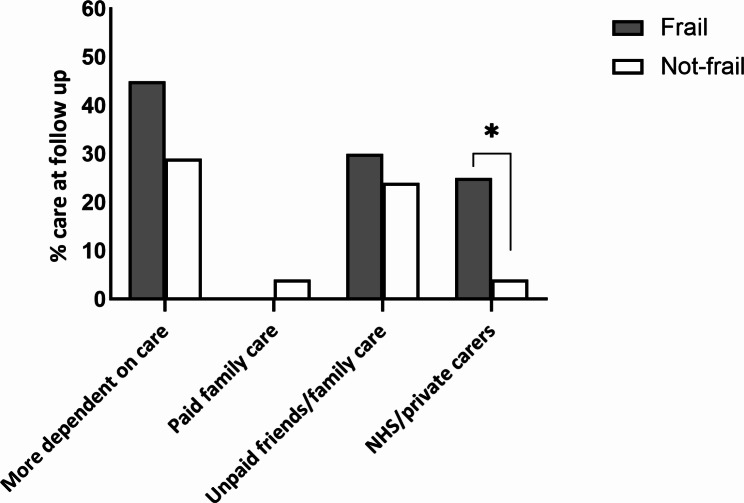
Care needs at follow up for frail and not-frail patients. ** NHS/private carers frail: 25% vs. not-frail: 4%, *p* < 0.01. (Fishers exact test)

At both discharge and follow up, frail patients reported more problems of any severity in all EQ5D dimensions except pain, compared to the not-frail cohort (Fig. 2A, B). Both groups of older patients reported increased self-care problems at follow up compared to discharge, but this was significantly higher in those with frailty (Frail: 88% vs. Not-frail: 55%, *p* = 0.01, Fig. 2B). Twice the proportion of frail patients were anxious or depressed at follow up compared to the not-frail group (Frail: 76% vs. Not-frail: 39%, *p* < 0.01, Fig. 2B). Although not significant, frail patients were more dependent on care at follow-up (Frail: 45% vs. Not-frail: 29%, *p* = 0.191) and there was a five-fold increase in requirements for state-funded or private carers in those with frailty (Frail: 25% vs. Not-frail: 3.5%, *p* < 0.01, Fig. 3).

## Discussion

This multi-site study examined survival and health related quality of life at six months after injury in older trauma patients with or without pre-injury frailty. Death was strongly associated with frailty during the six-month post discharge period. At six months, quality of life had deteriorated from discharge baseline for frail survivors but improved in those without frailty. Frail patients experienced more problems across all EQ-5D-5 L dimensions except pain and there was a significant increase in state-funded and private care provision associated with frailty.

Health related quality of life is an important outcome for older patients after critical illness or injury [29]. At six months post trauma frailty has been strongly associated with adverse functional status [11] and reduced neurological recovery [30]. Our frail trauma patients experienced poor quality of life at this time-point. HRQoL is reported to ‘recover’ by six months after hip fracture in the majority of older people [31] and in our not-frail cohort we observed an improvement in quality of life at this stage post injury. However, this was not the case for those with frailty, where HRQoL had significantly deteriorated from the discharge baseline. The six-month HRQoL scores for our frail cohort were worse than those of older trauma patients in a recent study of longer-term recovery [32], although our group were older on average (79 years compared to 73 years). We found that frailty, increased age and not being discharged to the usual place of residence were associated with poor HRQoL at six-months. Identifying frail trauma patients during the in-hospital phase of care and implementing frailty-specific pathways [33, 34] similar to those seen with hip-fracture populations [31], may enable targeted discharge planning, community rehabilitation and information to shape realistic expectations and prognostication [12, 35], none of which is standardardised UK trauma practice currently.

Self-care and anxiety and depression were the greatest problems experienced at six-months post injury for frail patients. Injury in older people can lead to a process of activity restrictions and isolation which negatively influences psychological quality of life [36]. The effects of frailty can also adversely affect engagement in usual activities [37, 38] which may also impact on mood and mental health. In hip fracture populations frailty on admission has been reported as a strong prognostic factor for depressive symptoms up to a year after injury [39]. Whilst frailty is a chronic state of low physiologic reserve, the effects of an acute event such as trauma may enhance progression in frail status [40] increasingly impairing psychological and physical quality of life. Post-discharge recovery programmes for older frail patients following acute hospital episodes have reported improvements in longer-term HRQoL [41], with major benefits for the self-care and usual activities dimensions within Eq. 5D [42]. Frailty-led targeted discharge planning and specialist community support may help to avoid or improve the problems reported by our frail trauma patients [43].

Frail patients had increased formal care needs at home similar to that of a larger trauma study where frailty led to discharge ‘home with health care’ [44]. Frailty is associated with increasing health and social care costs [45] and many older people have a strong preference for living as independently as possible with support within their own homes [46]. The challenge is identifying which community-based interventions are clinically and cost effective for frail older people [47], to support those living at home in improving or maintaining quality of life after significant injury.

### Limitations

This study has a number of limitations. Firstly, the proportion of frail patients alive and available for follow up in the longer term was small. There were no site-specific differences to account for the loss to follow-up, but it may be that some patients chose not to respond as they did not wish to be reminded of their traumatic event and hospitalisation. However, in those we could follow-up we believe that these findings provide a baseline for a larger scale investigation of quality of life and recovery in frail older trauma survivors. Secondly, we only used EQ-5D-5 L to measure and assess HRQoL. EQ-5D-5 L is a patient-reported outcome measure which assesses health status or HRQoL at specific points in time, which we were able to do, compared to population norms, which we were not. It may however be challenging to make comparisons between general population norms and older frail populations [48]. Despite this limitation, EQ-5D-5 L is a patient reported outcome measure recommended for used in older people [49] and recently identified as suitable for further validation with acute older frail populations [50]. Finally, we measured longer term HRQoL and outcome at six months post injury, whereas other trauma studies have followed up patients at time-points between three months and two-three years. We acknowledge that HRQoL may have improved for patients after the six-month period but given the deterioration seen in the frail group, it may also have worsened. Optimal time to measure HRQoL lacks consensus however a recent study of trauma patients aged ≥ 65 years showed no improvement in EQ5D-5 L HRQoL after six months post injury [32].

## Conclusion

Despite considerable loss to follow up, frailty in older trauma patients was statistically associated with post-discharge mortality, and in survivors, worse HRQoL and more physical and psychological problems at six months after injury. If frailty is a predictor of poor longer-term HRQoL, specialist pathways should be integral to trauma discharge planning for older patients, in order to set realistic goals and optimize the quality of post-discharge recovery.

## Data Availability

The datasets used and/or analysed during the current study are available from the corresponding author on reasonable request.

## Abbreviations

ANOVA: Analysis of Variance
CFS: Clinical Frailty Scale
HRQoL: Health Related Quality of Life
MTC: Major Trauma Centre
VAS: Visual Analogue Scale

## References

[1] Kozar RA, Arbabi S, Stein DM, Shackford SR, Barraco RD, Biffl WL, Brasel KJ, Cooper Z, Fakhry SM, Livingston D (2015). Injury in the aged: geriatric trauma care at the crossroads. J Trauma Acute care Surg.

[2] Zhao F, Tang B, Hu C, Wang B, Wang Y, Zhang L (2020). The impact of frailty on posttraumatic outcomes in older trauma patients: a systematic review and meta-analysis. J Trauma Acute care Surg.

[3] TARN. : Major trauma in older people. https://www.tarnacuk/Contentaspx?c=3793 2017.

[4] Management of Older Major Trauma Patients. (Third Edition) [https://www.c4ts.qmul.ac.uk/downloads/2021-pan-london-major-trauma-system-management-of-older-trauma.-third-editionapril-2021.pdf].

[5] Bardes JM, Benjamin E, Schellenberg M, Inaba K, Demetriades D (2019). Old Age with a traumatic mechanism of Injury should be a Trauma Team Activation Criterion. J Emerg Med.

[6] Eagles D, Godwin B, Cheng W, Moors J, Figueira S, Khoury L, Fournier K, Lampron J (2020). A systematic review and meta-analysis evaluating geriatric consultation on older trauma patients. J Trauma Acute care Surg.

[7] Rickard F, Ibitoye S, Deakin H, Walton B, Thompson J, Shipway D, Braude P (2021). The clinical Frailty Scale predicts adverse outcome in older people admitted to a UK major trauma centre. Age Ageing.

[8] Pecheva M, Phillips M, Hull P, Carrothers AO, Queally JM (2020). The impact of frailty in major trauma in older patients. Injury.

[9] Carter B, Short R, Bouamra O, Parry F, Shipway D, Thompson J, Baxter M, Lecky F, Braude P (2022). A national study of 23 major trauma centres to investigate the effect of frailty on clinical outcomes in older people admitted with serious injury in England (FiTR 1): a multicentre observational study. Lancet Healthy Longev.

[10] Joseph B, Orouji Jokar T, Hassan A, Azim A, Mohler MJ, Kulvatunyou N, Siddiqi S, Phelan H, Fain M, Rhee P (2017). Redefining the association between old age and poor outcomes after trauma: the impact of frailty syndrome. J Trauma Acute care Surg.

[11] Maxwell CA, Mion LC, Mukherjee K, Dietrich MS, Minnick A, May A, Miller RS (2016). Preinjury physical frailty and cognitive impairment among geriatric trauma patients determine postinjury functional recovery and survival. J Trauma Acute care Surg.

[12] Maxwell CA, Patel MB, Suarez-Rodriguez LC, Miller RS (2019). Frailty and Prognostication in geriatric Surgery and trauma. Clin Geriatr Med.

[13] Hamidi M, Zeeshan M, Leon-Risemberg V, Nikolich-Zugich J, Hanna K, Kulvatunyou N, Saljuqi AT, Fain M, Joseph B (2019). Frailty as a prognostic factor for the critically ill older adult trauma patients. Am J Surg.

[14] Geraerds A, Richardson A, Haagsma J, Derrett S, Polinder S (2020). A systematic review of studies measuring health-related quality of life of general injury populations: update 2010–2018. Health Qual Life Outcomes.

[15] Aljeaidi MS, Haaksma ML, Tan ECK (2022). Polypharmacy and trajectories of health-related quality of life in older adults: an Australian cohort study. Qual Life Res.

[16] TARN.: Running Major Trauma PROMS. https://www.tarnacuk/content/downloads/53/PROMs%20Guidance%20Document2021pdf 2018

[17] Marten O, Brand L, Greiner W (2022). Feasibility of the EQ-5D in the elderly population: a systematic review of the literature. Qual Life Res.

[18] Santino C, Zeeshan M, Hamidi M, Hanna K, Saljuqi AT, Kulvatunyou N, Haddadin Z, Northcutt A, Joseph B (2019). Prospective evaluation of health-related quality of life in geriatric trauma patients. Surgery.

[19] Jarman H, Crouch R, Baxter M, Wang C, Peck G, Sivapathasuntharam D, Jennings C, Cole E (2021). Feasibility and accuracy of ED frailty identification in older trauma patients: a prospective multi-centre study. Scand J Trauma Resusc Emerg Med.

[20] Eurolqol.org. EQ-5D-5L. https://euroqolorg/eq-5d-instruments/eq-5d-5l-about/. 2021

[21] Fleischhacker E, Trentzsch H, Kuppinger D, Meigel F, Beyer F, Hartl WH (2018). Long-term changes of patient-reported quality of life after major trauma: the importance of the time elapsed after injury. Injury.

[22] Christensen MC, Banner C, Lefering R, Vallejo-Torres L, Morris S (2011). Quality of life after severe trauma: results from the global trauma trial with recombinant factor VII. J Trauma.

[23] Lutomski JE, Krabbe PF, Bleijenberg N, Blom J, Kempen GI, MacNeil-Vroomen J, Muntinga ME, Steyerburg E, Olde-Rikkert MG, Melis RJ (2017). Measurement properties of the EQ-5D across four major geriatric conditions: findings from TOPICS-MDS. Health Qual Life Outcomes.

[24] Loggers SAI, Willems HC, Van Balen R, Gosens T, Polinder S, Ponsen KJ, Van de Ree CLP, Steens J, Verhofstad MHJ, Zuurmond RG et al. Evaluation of quality of Life after Nonoperative or Operative Management of proximal femoral fractures in Frail Institutionalized patients: the FRAIL-HIP study. JAMA Surg 2022. 10.1001/jamasurg.2022.0089 PMC8892372 35234817

[25] Devlin NJ, Brooks R (2017). EQ-5D and the EuroQol Group: past, Present and Future. Appl Health Econ Health Policy.

[26] Maxwell CA, Dietrich MS, Minnick AF, Mion LC (2015). Preinjury physical function and frailty in injured older adults: self- Versus Proxy responses. J Am Geriatr Soc.

[27] Carpenter JR, Smuk M (2021). Missing data: a statistical framework for practice. Biom J.

[28] Rockwood K, Song X, MacKnight C, Bergman H, Hogan DB, McDowell I, Mitnitski A (2005). A global clinical measure of fitness and frailty in elderly people. CMAJ: Can Med Association J = J de l’Association medicale canadienne.

[29] Bagshaw SM, Stelfox HT, Johnson JA, McDermid RC, Rolfson DB, Tsuyuki RT, Ibrahim Q, Majumdar SR (2015). Long-term association between frailty and health-related quality of life among survivors of critical Illness: a prospective multicenter cohort study. Crit Care Med.

[30] Galimberti S, Graziano F, Maas AIR, Isernia G, Lecky F, Jain S, Sun X, Gardner RC, Taylor SR, Markowitz AJ (2022). Effect of frailty on 6-month outcome after traumatic brain injury: a multicentre cohort study with external validation. Lancet Neurol.

[31] Peeters CM, Visser E, Van de Ree CL, Gosens T, Den Oudsten BL, De Vries J (2016). Quality of life after hip fracture in the elderly: a systematic literature review. Injury.

[32] Freigang V, Müller K, Ernstberger A, Kaltenstadler M, Bode L, Pfeifer C, Alt V, Baumann F. Reduced Recovery Capacity after Major Trauma in the Elderly: results of a prospective Multicenter Registry-based Cohort Study. J Clin Med 2020, 9(8). 10.3390/jcm9082356 PMC7464491 32717963

[33] Bryant EA, Tulebaev S, Castillo-Angeles M, Moberg E, Senglaub SS, O’Mara L, McDonald M, Salim A, Cooper Z (2019). Frailty Identification and Care Pathway: an Interdisciplinary Approach to care for older trauma patients. J Am Coll Surg.

[34] Engelhardt KE, Reuter Q, Liu J, Bean JF, Barnum J, Shapiro MB, Ambre A, Dunbar A, Markzon M, Reddy TN (2018). Frailty screening and a frailty pathway decrease length of stay, loss of independence, and 30-day readmission rates in frail geriatric trauma and emergency general Surgery patients. J Trauma Acute care Surg.

[35] Patel V, Lindenmeyer A, Gao F, Yeung J (2023). A qualitative study exploring the lived experiences of patients living with mild, moderate and severe frailty, following hip fracture Surgery and hospitalisation. PLoS ONE.

[36] Zidén L, Scherman MH, Wenestam CG (2010). The break remains – elderly people’s experiences of a hip fracture 1 year after discharge. Disabil Rehabil.

[37] Rand D, Sternberg SA, Gasner Winograd R, Buckman Z, Bentur N. The contribution of Frailty to participation of older adults. Int J Environ Res Public Health 2022, 19(3). 10.3390/ijerph19031616 PMC8835014 35162637

[38] Koizia L, Kings R, Koizia A, Peck G, Wilson M, Hettiaratchy S, Fertleman MB (2019). Major trauma in the elderly: Frailty decline and patient experience after injury. Trauma.

[39] van de Ree CLP, de Munter L, Biesbroeck BHH, Kruithof N, Gosens T, de Jongh MAC (2020). The prevalence and prognostic factors of psychological distress in older patients with a hip fracture: a longitudinal cohort study. Injury.

[40] Muscedere J, Waters B, Varambally A, Bagshaw SM, Boyd JG, Maslove D, Sibley S, Rockwood K (2017). The impact of frailty on intensive care unit outcomes: a systematic review and meta-analysis. Intensive Care Med.

[41] Senior HE, Parsons M, Kerse N, Chen MH, Jacobs S, Hoorn SV, Anderson CS (2014). Promoting independence in frail older people: a randomised controlled trial of a restorative care service in New Zealand. Age Ageing.

[42] Comans TA, Peel NM, Gray LC, Scuffham PA (2013). Quality of life of older frail persons receiving a post-discharge program. Health Qual Life Outcomes.

[43] Turner G, Clegg A (2014). Best practice guidelines for the management of frailty: a British Geriatrics Society, Age UK and Royal College of General Practitioners report. Age Ageing.

[44] Hatcher VH, Galet C, Lilienthal M, Skeete DA, Romanowski KS (2019). Association of Clinical Frailty scores with Hospital Readmission for Falls after Index Admission for Trauma-Related Injury. JAMA Netw Open.

[45] Han L, Clegg A, Doran T, Fraser L (2019). The impact of frailty on healthcare resource use: a longitudinal analysis using the Clinical Practice Research Datalink in England. Age Ageing.

[46] van Leeuwen KM, van Loon MS, van Nes FA, Bosmans JE, de Vet HCW, Ket JCF, Widdershoven GAM, Ostelo R (2019). What does quality of life mean to older adults? A thematic synthesis. PLoS ONE.

[47] Crocker TF, Clegg A, Riley RD, Lam N, Bajpai R, Jordão M, Patetsini E, Ramiz R, Ensor J, Forster A (2021). Community-based complex interventions to sustain independence in older people, stratified by frailty: a protocol for a systematic review and network meta-analysis. BMJ Open.

[48] Hartholt KA, van Beeck EF, Polinder S, van der Velde N, van Lieshout EM, Panneman MJ, van der Cammen TJ, Patka P (2011). Societal consequences of falls in the older population: injuries, healthcare costs, and long-term reduced quality of life. J Trauma.

[49] Peden CJ, Grocott MP (2014). National Research Strategies: what outcomes are important in peri-operative elderly care?. Anaesthesia.

[50] van Oppen JD, Alshibani A, Coats TJ, Graham B, Holch P, Lalseta J, Mackintosh N, Richardson V, Riley P, Valderas JM (2022). A systematic review and recommendations for prom instruments for older people with frailty in emergency care. J Patient Rep Outcomes.

